# Ultrasound scoring system for prenatal diagnosis of placenta accreta spectrum

**DOI:** 10.1186/s12884-023-05886-x

**Published:** 2023-08-07

**Authors:** Junling Zhang, Hezhou Li, Demin Feng, Juan Wu, Zhaoyu Wang, Fan Feng

**Affiliations:** The Third Affiliated Hospital of Zhengzhou University, Zhengzhou, China

**Keywords:** Placenta accreta spectrum, Abnormally invasive placenta, Accreta, Ultrasound scoring system, Prenatal diagnosis

## Abstract

**Background:**

To develop an ultrasound scoring system for placenta accreta spectrum (PAS), evaluate its diagnostic value, and provide a practical approach to prenatal diagnosis of PAS.

**Methods:**

A total of 532 pregnant women (n = 184 no PAS, n = 120 placenta accreta, n = 189 placenta increta, n = 39 placenta percreta) at high-risk for placenta accreta who delivered in the Third Affiliated Hospital of Zhengzhou University between January 2021 and December 2022 underwent prenatal ultrasound to evaluate placental invasion. An ultrasound scoring system that included placental and cervical morphology and history of cesarean section was created. Each feature was assigned a score of 0 ~ 2, according to severity. Thresholds for the total ultrasound score that discriminated between no PAS, placenta accreta, placenta increta, and placenta percreta were calculated.

**Results:**

Univariate and multivariate regression analysis identified seven indicators of PAS that were included in the ultrasound scoring system, including placental location, placental thickness, presence/absence of the retroplacental space, thickness of the retroplacental myometrium, presence/absence of placental lacunae, retroplacental myometrial blood flow and history of cesarean section. Using the final ultrasound scoring system, no PAS is diagnosed at a total score < 5, placenta accreta or placenta increta is diagnosed at a total score 5–10, and placenta percreta is diagnosed at a total score ≥ 10.

**Conclusions:**

This study identified seven indicators of PAS and included them in an ultrasound scoring system that has good diagnostic efficacy and clinical utility.

**Trial registration:**

ChiCTR2300069261 (retrospectively registered on 10/03/2023).

## Background

Placenta accreta spectrum (PAS) refers to abnormal adhesion or invasion of trophoblastic tissue into the myometrium [1–4]. PAS can cause maternal morbidity such as uterine rupture, severe postpartum hemorrhage, multiorgan failure, and preterm birth [3, 5]. Population based studies indicate the incidence of PAS is increasing by 30% every 10 years [5]. Among pregnant women (> 12 weeks of gestation) attending the Third Affiliated Hospital of Zhengzhou University in Henan province, China, the prevalence of PAS was 32.9 per 1,000 (361/10,956) in 2015, and 34.9 per 1,000 (452/12,946) in 2021, and higher than the prevalence of PAS on mainland China (0.22%) [6]. Notably, the incidence of PAS at the Third Affiliated Hospital of Zhengzhou University is high, likely because this center is a provincial maternal and child medical center attended by high-risk pregnant women.

Ultrasound is the preferred screening tool for PAS, and can reduce obstetric morbidity among at-risk women [3, 7]. Ultrasound features, including loss of the normal retroplacental space, myometrial thinning, placental lacunae, and hypervascularity of the uterine serosa bladder wall, contribute to the prenatal diagnosis of PAS [8–11]. Identifying women with PAS allows multidisciplinary case management in a tertiary maternity care center and decreased maternal morbidity [1, 7, 11–13]. At our center, despite the increased prevalence of PAS from 2015 to 2021, the number of hysterectomies due to PAS decreased (5/361 in 2015 vs. 2/452 in 2021), and there were no deaths.

Due to the varying degrees of placental invasion (placenta accreta, placenta increta, and placenta percreta), the ultrasound features of PAS are complex and diverse, making an accurate diagnosis difficult. Evidence suggests that one-half to two-thirds of PAS cases remain undiagnosed before delivery [14, 15], including approximately one-third of PAS cases in specialist centers [16]. The sensitivity and specificity of various ultrasound features for PAS change across the spectrum of placental invasion [8, 17], and there is significant interobserver variability in the interpretation of placental invasion [8, 18] as most ultrasound features are poorly defined [19]. At present, there is no consensus on a diagnostic standard for PAS. Combined evaluation of multiple indicators [9, 20–25] can objectively assess risk of PAS. The “Placenta Accreta Index” [9, 21], “ultrasound staging system for PAS” [1], and “two-criteria system” [11] have good diagnostic performance for PAS; however, sample selection (placenta previa or a history of cesarean section, or both), and the varying number, selection and assignment of scoring indicators limit their clinical application. Although novel ultrasound features have been proposed [26], accurate prenatal diagnosis of PAS is challenging, especially in less severe cases, and diagnostic criteria remain under debate. The objective of this study was to prospectively develop an ultrasound scoring system for PAS, evaluate its diagnostic value, and provide a practical approach to prenatal diagnosis of PAS.

## Methods

This was a double-blind prospective study. A total of 532 pregnant women who delivered in the Third Affiliated Hospital of Zhengzhou University between January 2021 and December 2022 underwent ultrasound to evaluate placental invasion. Inclusion criteria were: (1) high-risk for placenta accreta [3, 11, 17, 27–29] due to history of surgery (cesarean section, uterine myomectomy, labor induction, uterine curettage, induced abortion), placenta previa, primary uterine abnormalities (bicornuate uterus, adenomyosis, submucosal myoma), smoking, and/or advanced age; (2) gestational age ≥ 28 weeks; and (3) singleton pregnancy. Exclusion criteria were (1) serious diseases of the heart, brain, liver, kidney and other organs; or (2) abnormal coagulation function or malignant tumors. This study was reviewed and approved by the Medical Ethics Committee of the Third Affiliated Hospital of Zhengzhou University. All women provided informed consent before ultrasonic assessment.

Women were evaluated by transabdominal ultrasound (Voluson E8, GE Medical Systems, Zipf, Austria) using a system equipped with a 4-to 8-MHz transducer. Transvaginal ultrasound is the gold standard for diagnosis of a Cesarean scar pregnancy in early pregnancy, and is superior to transabdominal ultrasound. However, transvaginal ultrasound has limited utility for PAS in late pregnancy, as direction of the beam and field of vision is limited to the cervix and lower portion of the uterus. In late pregnancy, abdominal ultrasound is advantageous.

A full bladder was required to clearly visualize the lower anterior uterine wall. Placental location, placental thickness, presence/absence of the retroplacental space, thickness of the retroplacental myometrium, bladder line interruption, presence/absence of placental lacunae, retroplacental myometrial blood flow, presence/absence of a cervical sinus, and cervical morphology were observed, and history of cesarean section was recorded. To measure placental thickness and thickness of the retroplacental myometrium, the probe was positioned so the beam was perpendicular to the uterine wall. Placental thickness was measured at the thickest part. When the retroplacental myometrium was measured, the image was enlarged so the hypoechoic muscle layer behind the placenta could be measured to obtain the smallest myometrial thickness in the sagittal plane. Increasing retroplacental myometrial blood flow was defined based on color Doppler ultrasound performed with a full bladder, and a blood flow velocity ≥ 20 cm/s. In the sagittal plane, normal blood flow appeared scattered, with a discontinuous distribution in the uterine wall behind the placenta, or as a regular, straight, thin strip of uniform color, representing a blood vessel running along the uterine wall. Increased blood flow is due to thickened and tortuous blood vessels, which appeared as multicolored, overlapping blood vessels that crisscrossed, or as turbulent blood flow along the uterine wall. A scoring system was created where each feature was assigned a score between 0 and 2 (Table 1) [22, 23].

**Table 1:** Preliminary ultrasound scoring system for PAS

Feature	0	1	2
Placental location	Normal	Low-lying placenta (≤ 2 cm)	Placenta previa
Placental thickness	≤ 30 mm	30 mm ∼50 mm	≥ 50 mm
Retroplacental space	Present	Absent	/
Thickness of the retroplacental myometrium	> 1 mm	≤ 1 mm	Absence
Bladder line interruption	Normal	Interrupt	Absence and placental bulge
Placental lacunae	None	Present	Numerous and confluent
Retroplacental myometrial blood flow	Normal	Increased	Numerous and confluent
Cervical sinus	None	Present	Numerous and confluent
Cervical morphology	Normal	Incomplete	Disappeared
History of cesarean section	None	1	≥ 2

Women’s medical records were reviewed after delivery. Maternal age, gestational age at delivery, intraoperative blood loss, degree of placental invasion, implantation site, and pathology were recorded. Women were divided into 4 groups: no PAS, placenta accreta, placenta increta, and placenta percreta. Obstetricians were blinded to the results of the scoring system conducted by the authors (J.Z, J.W, Z.W and F.F). All statistical analysis were then performed by D.F.

SPSS v26.0 was used for statistical analysis. Normally distributed continuous variables are reported as mean ± standard deviation, and were compared with one-way analysis of variance. Non-normally distributed continuous variables are reported as median (Q1, Q3), and were compared with the Kruskal-Wallis H test. Categorical variables are expressed as frequency and percentage, and were compared with the χ2 test or Fisher exact test. Binary logistic regression analysis was used to calculate odds ratios (ORs) and 95% confidence intervals (95% CIs) to describe the associations between the features of the ultrasound scoring system and the degree of placental invasion. Meaningful features were selected and included in a final scoring system to calculate a total score. Receiver operating characteristic (ROC) curves were used to calculate the thresholds for the total score that discriminated between no PAS, placenta accreta, placenta increta, and placenta percreta. P < 0.05 was considered statistically significant.

## Results

### Post-partum follow-up data

The pregnant women included in this study (n = 532) were aged 20 to 50 years, and gestational age at delivery was 28 to 40.5 weeks.

After delivery, 184 patients had no PAS and 348 patients had PAS. Among those with PAS, 120 women (34.5%) had placenta accreta, 189 women (54.3%) had placenta increta, and 39 women (11.2%) had placenta percreta. There were significant differences in gestational age at delivery and intraoperative blood loss among women with no PAS, women with placenta accreta, women with placenta increta and women with placenta percreta, but there was no significant difference in maternal age (Table 2).

**Table 2:** Maternal age, gestational age at delivery, and intraoperative blood loss

	N	Maternal age	Gestational age at delivery	Intraoperative blood loss
		*[M(Q1, Q3), y]*	*[M(Q1, Q3),w]*	*[M(Q1, Q3), ml]*
No PAS	184	32(29,37)	37.35(35.70,38.48)	300(300,400)
Accreta	120	33(31,36)	36.60(35.43,37.5)	500(300,800)
Increta	189	33(31,36)	36.30(35.40,37.20)	1000(600,1600)
Percreta	39	33(31,35)	36.10(35.20,36.40)	2500(1500,3000)
*P*		0.762	P<0.001	P<0.001

Among the study population, 13 women (2.4%) delivered vaginally and 519 women (97.6%) delivered by cesarean Sect. 246 women (46.2%) underwent abdominal aortic balloon occlusion, and 307 women (57.7%) underwent uterine artery ligation, uterine tamponade, balloon compression and other measures for hemostasis. Among those with placenta percreta, 7 women required bladder repair and 3 women required a hysterectomy.

Causes of premature delivery in the study population were hemorrhage due to placenta previa, placental abruption, premature rupture of membranes, preterm uterine contractions, and PAS. The study included one woman aged 50 years. The woman had a low-lying placenta, placenta accreta, velamentous cord insertion and vasa previa. She delivered by cesarean section at 33 weeks of gestation, and intraoperative blood loss was 300ml. Of note, three women with placenta percreta delivered late (2 women delivered at 38 weeks of gestation and 1 woman delivered at 39 weeks of gestation), because they were likely from rural areas without access to standard perinatal care. These women had a history of 0–4 cesarean sections.

### Logistic regression analysis

The variable assignment method for binary logistic regression is shown in Table 3. Dependent variables were no PAS (n = 184) and PAS (n = 348). Independent variables were placental location, placental thickness, presence/absence of the retroplacental space, thickness of the retroplacental myometrium, bladder line interruption, presence/absence of placental lacunae, retroplacental myometrial blood flow, presence/absence of a cervical sinus, cervical morphology, and history of cesarean section (Table 3). The Hosmer and Lemeshow goodness of fit test for logistic regression showed that the model was correctly specified (*p* = 0.470).

**Table 3:** Ultrasound scoring system for PAS: variable assignment method for binary logistic regression

Variables	Assignment
Dependent variable	“1” for PAS, “0” for no PAS
*Independent variables*	
Placental location	“1” for Placenta previa or low-lying,“0” for Normal
Placental thickness	“1” for >30 mm, “0” for ≤ 30 mm
Retroplacental space	“1” for Absence, “0” for Present
Thickness of retroplacental myometrium	“1” for ≤ 1 mm or Absence, “0” for >1 mm
Bladder line interruption	“1” for Interrupt, “0” for Normal
Placental lacunae	“1” for Present, “0” for None
Retroplacental myometrial blood flow	“1” for Increased, “0” for Normal
Cervical sinus	“1” for Present, “0” for None
Cervical morphology	“1” for Incomplete or Disappeared, “0” for Normal
History of cesarean section	“1” for ≥ 1, “0” for None

Logistic regression showed significant associations of placental location, placental thickness, presence/absence of the retroplacental space, thickness of the retroplacental myometrium, presence/absence of placental lacunae, retroplacental myometrial blood flow and history of cesarean section with PAS, but no significant associations of presence/absence of a cervical sinus, cervical morphology, and bladder line interruption with PAS (Table 4). Placental location was the most important indicator of PAS, followed by history of cesarean section and presence/absence of placental lacunae. Placenta previa increased the risk of PAS 14.11 times compared to normal placental location.

**Table 4:** Binary logistic regression analysis

Feature	OR	95% CI		*P*
		Lower	Upper	
Placental location	14.110	6.836	29.123	<0.001
Placental thickness	2.027	1.129	3.640	0.018
Retroplacental space	3.005	1.728	5.226	<0.001
Thickness of the retroplacental myometrium	2.083	1.146	3.785	0.016
Bladder line interruption	104967577.100	0.000	.	0.999
Placental lacunae	3.685	1.163	11.675	0.027
Retroplacental myometrial blood flow	1.795	1.068	3.020	0.027
Cervical sinus	18497277.377	0.000	.	0.998
Cervical morphology	9.164	0.875	95.949	0.064
History of cesarean section	4.764	2.731	8.311	<0.001

### Ultrasound scoring system for PAS

**Fig. 1:**
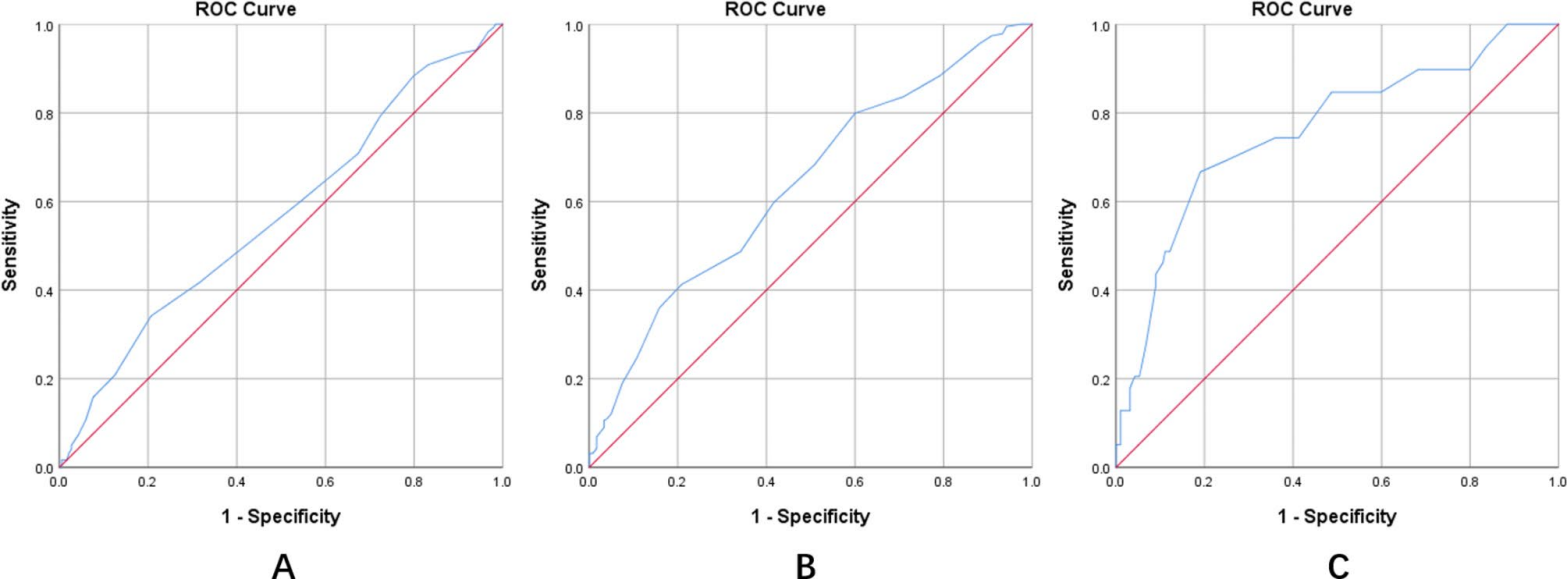
ROC curves for placental thickness **(A)** No PAS vs. placenta accreta, AUC = 0.568, 95% CI: 0.502–0.634. **(B)** Placenta accreta vs. increta, AUC = 0.636, 95% CI: 0.574–0.699. **(C)** Placenta increta vs. percreta, AUC = 0.760, 95%CI: 0.672–0.848. AUC: Area under curve; CI: confidence interval

ROC curves were used to determine thresholds of placental thickness that discriminated no PAS, placenta accreta, placenta increta, and placenta percreta. Findings showed no PAS was diagnosed at a placental thickness ≤ 35 mm, placenta accreta and placenta increta were diagnosed at a placental thickness of 35 − 40 mm, and placenta percreta was diagnosed at a placental thickness ≥ 40 mm (Table 5) (Fig. 1).

**Table 5:** ROC curve analysis for placental thickness

	AUC	95%CI		Maximum Yoden index	Cut-off point of placental thickness
		Lower	Upper		
No PAS vs. placenta accreta	0.568	0.502	0.634	1.135	35.5
Placenta accreta vs. increta	0.636	0.574	0.699	1.205	36.5
Placenta increta vs. percreta	0.760	0.672	0.848	1.477	39.5

**Fig. 2:**
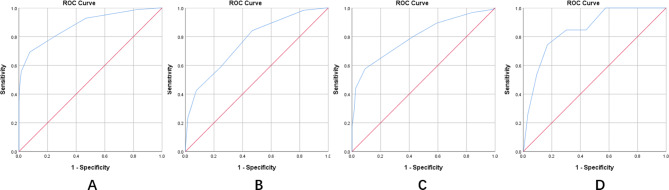
ROC curves for the total score of the final ultrasound scoring system for PAS **(A)** No PAS /PAS, AUC = 0.880, 95% CI: 0.852–0.908; **(B)** No PAS/Accreta, AUC = 0.766, 95% CI: 0.712–0.820; **(C)** Accreta /Increta, AUC = 0.799, 95% CI: 0.751–0.847; D: AUC = 0.848, 95% CI: 0.788–0.909

The final ultrasound scoring system for PAS is shown in Table 6. ROC curves were used to determine thresholds for the total score that discriminated no PAS, placenta accreta, placenta increta, and placenta percreta. Findings showed no PAS was diagnosed at a total score < 3 points, placenta accreta was diagnosed at a total score ≥ 3 points (sensitivity 84%, specificity 53%), PAS was diagnosed at a total score ≥ 5 (sensitivity 69%, specificity 92%), placenta increta was diagnosed at a total score ≥ 7 points (sensitivity 58%, specificity 91%), and placenta percreta was diagnosed at total score ≥ 10 (sensitivity 74%, specificity 83%). (Table 7) (Fig. 2).

**Table 6:** Final ultrasound scoring system for PAS

Feature	0	1	2
Placental location	Normal	Low-lying (≤ 2 cm)	Previa
Placental thickness	≤ 35 mm	35 ~ 40 mm	≥ 40 mm
Retroplacental space	Present	Absence	/
Thickness of the retroplacental myometrium	> 1 mm	≤ 1 mm	Absence
Placental lacunae	None	Present	Numerous and confluent
Retroplacental myometrial blood flow	Normal	Hypervascularity	Numerous and confluent
History of cesarean section	0	1	≥ 2

**Table 7:** ROC curve analysis for the total score of the final ultrasound scoring system for PAS

	AUC	95%CI		Maximum Yoden index	Cut-off point of total score	Sensitivity	specificity
		Lower Bound	Upper Bound				
No PAS /PAS	0.880	0.852	0.908	1.617	5	69%	92%
No PAS/ Accreta	0.766	0.712	0.820	1.375	3	84%	53%
Accreta /Increta	0.799	0.751	0.847	1.485	7	58%	91%
Increta/Percreta	0.848	0.788	0.909	1.575	10	74%	83%

**Fig. 3:**
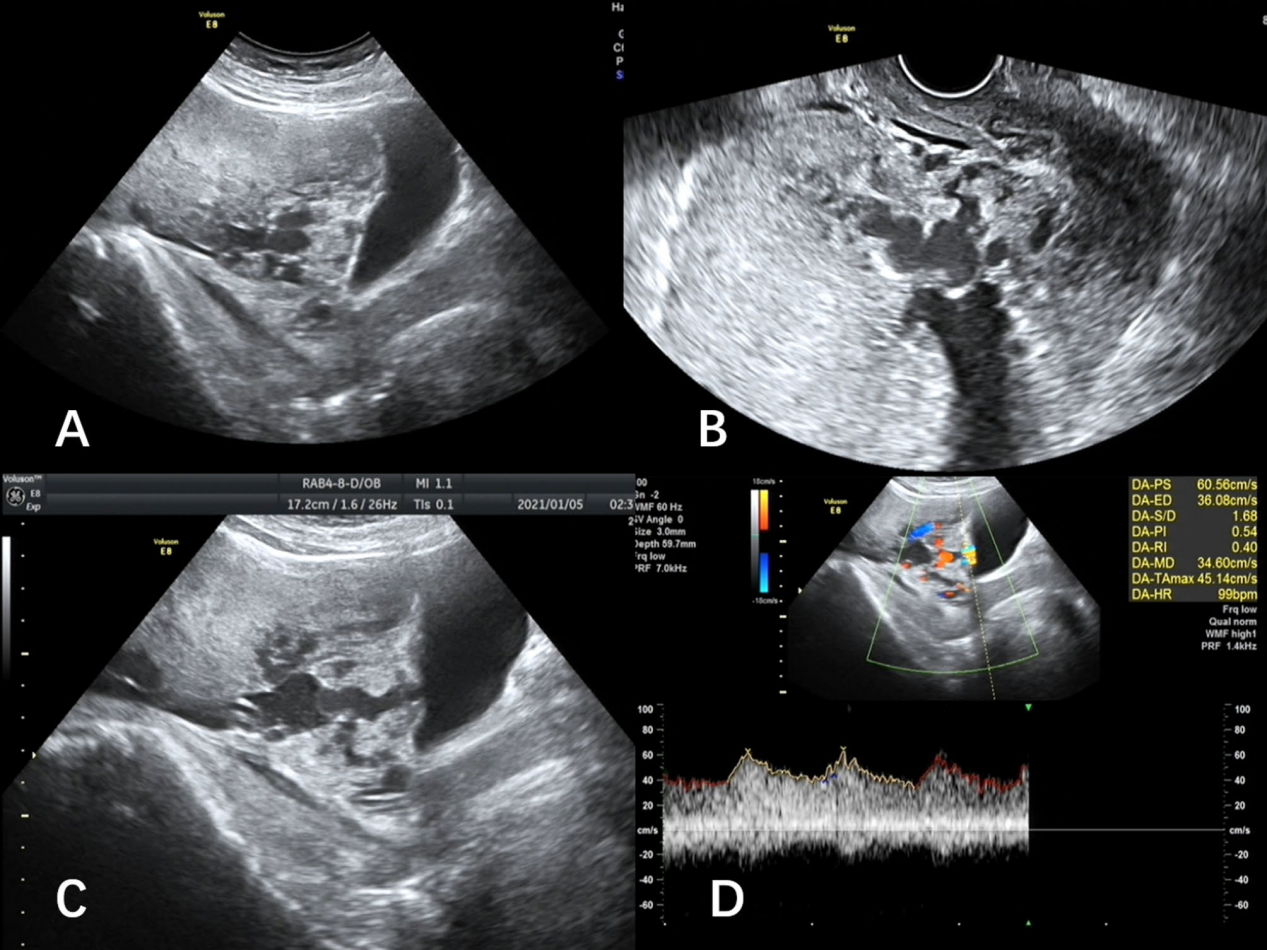
31y, G2P1. **A.** The woman had one previous cesarean Sect. (1 point). placenta previa (1 point), loss of the retroplacental space (1 point), and absent retroplacental myometrium (2 points). **B**. Transvaginal ultrasound. **C.** Numerous and confluent lacunae (2 points), and feeder vessels extending to the inferior anterior uterine wall. Color-doppler imaging shows numerous and confluent blood flow in the lacunae and retroplacental myometrium (2 points). The flow velocity of the arcuate artery reached 60 cm/s. **D**. Placental thickness is 48 mm (2 points). Total score is 11. The woman underwent cesarean section at 32 weeks of gestation. Balloon occlusion of the abdominal aorta and bilateral uterine artery embolization were performed. The placenta had penetrated the uterine wall and reached the posterior wall of the bladder. Intraoperative bleeding was 3000 ml. Bladder repair was performed

**Fig. 4:**
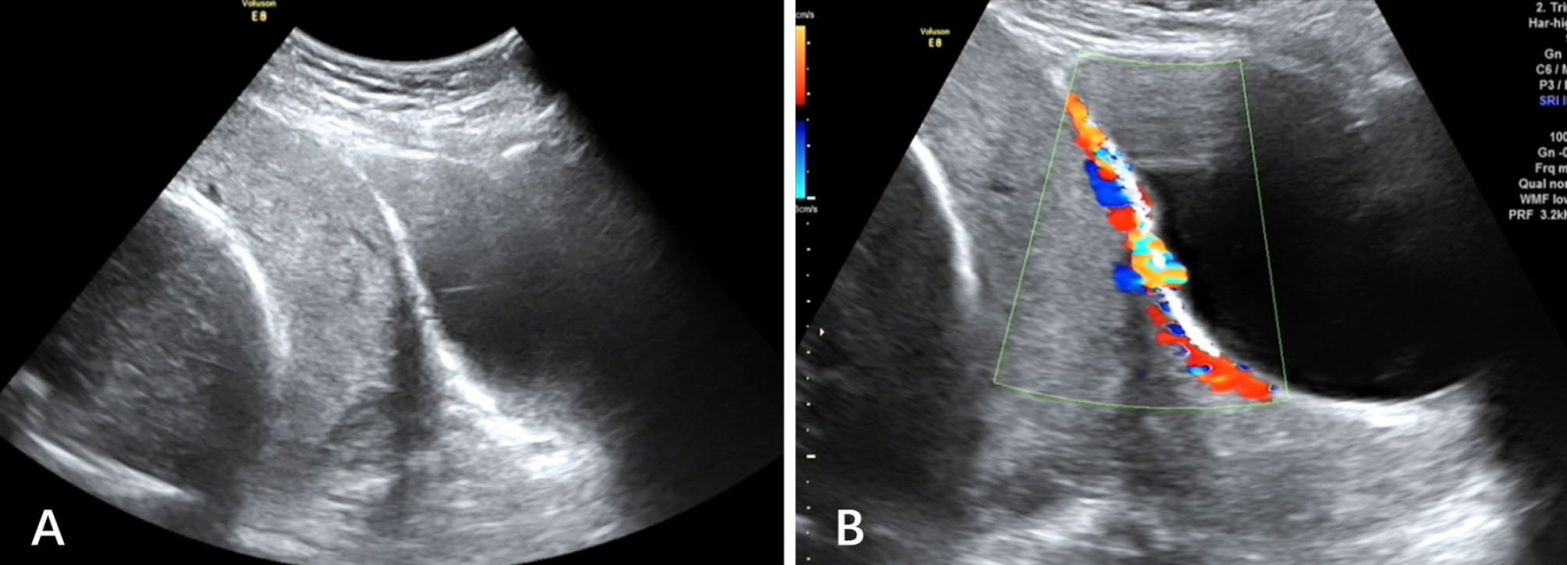
36y, G2P0, 1 miscarriage, no previous cesarean Sect. (0 point). **(A)** Placenta previa (2 points), loss of the retroplacental space (1 point), retroplacental myometrium thickness ≤ 1 mm (1 point). **(B)** Retroplacental myometrial blood flow was defined as hypervascularity (1 point); there were no placental lacunae (0 points); placental thickness was 35 mm (0 points). Total score was 5. The woman underwent cesarean section at 36 weeks, and the abdominal aorta was temporarily blocked by a balloon. Placenta increta was confirmed during the operation, and intraoperative bleeding was 1500 ml

To ensure our ultrasound scoring system provides a practical approach to prenatal diagnosis of PAS, we defined no PAS as a total score < 5, placenta accreta or placenta increta as a total score 5–10, and placenta percreta as a total score ≥ 10 (Figs. 3 and 4). These thresholds gave a false positive rate of 7.6% (14/184) in women with no PAS and a false negative rate of 30.7% (107/348) in women with PAS (69/107 in women with placenta accreta, 38/107 in women with placenta increta). Among the false negatives, 15.0%(16/107) women had intraoperative bleeding of 1000–2000 ml, and 1.9%(2/107) women had intraoperative bleeding > 2000 ml (2600 ml and 2700 ml).

## Discussion

This single center double-blind study developed an ultrasound scoring system for PAS, evaluated its diagnostic value, and provides a practical approach to prenatal diagnosis of PAS. Univariate and multivariate regression analysis identified seven indicators of PAS that were included in the ultrasound scoring system, including placental location, placental thickness, presence/absence of the retroplacental space, thickness of the retroplacental myometrium, presence/absence of placental lacunae, retroplacental myometrial blood flow and history of cesarean section. Using the final ultrasound scoring system, no PAS was diagnosed at a total score < 3 points, placenta accreta was diagnosed at a total score ≥ 3 points (sensitivity 84%, specificity 53%), PAS was diagnosed at a total score ≥ 5 (sensitivity 69%, specificity 92%), placenta increta was diagnosed at a total score ≥ 7 points (sensitivity 58%, specificity 91%), and placenta percreta was diagnosed at total score ≥ 10 (sensitivity 74%, specificity 83%). As anatomical and hemodynamic differences between placenta accreta and mild placenta increta are relatively slight, these conditions are difficult to distinguish on ultrasound images. Considering the poor sensitivity and specificity of thresholds < 3 points, ≥ 3 points and ≥ 7 points, clinical decision making, and implications for prognosis, interpretation of the score was simplified, such that no PAS is diagnosed at a total score < 5, placenta accreta or placenta increta is diagnosed at a total score 5–10, and placenta percreta is diagnosed at a total score ≥ 10. Using this score, clinicians can decide whether to terminate or prolong a pregnancy.

Combined evaluation of multiple features is likely to improve the accuracy of ultrasound diagnosis of PAS. Previous studies have developed other scoring systems, but these studies included women with ≥ 1 prior cesarean delivery or suspected morbidly adherent placenta on previous sonographic examination [20], pregnant women with persistent placenta previa [11], or pregnant women with ≥ 1 prior cesarean delivery and placenta previa or low-lying placenta [21, 22, 25], which affected the cut-off score. The different studies selected different features. In the present study, the scoring system used an objective and reasonable approach. Initially 10 features were considered, and after logistic regression and receiver operating characteristic (ROC) curve analysis, 7 meaningful PAS-related features were selected. The number of features other scoring systems used varied from 5 to 10 [20–22, 25]. The score is likely to be higher with more features especially for patients with placenta percreta and severe placenta increta, as these placentae will present more typical ultrasound manifestations of PAS.

Prenatal diagnosis of PAS by ultrasound is mainly based on gray-scale and color-doppler features [18, 30]. Multiparametric prediction models integrating imaging signs and pregnancy characteristics, such as the number of previous CS, can predict PAS more accurately than imaging alone [30]. In accordance with our findings, previous reports have recognized placenta previa and history of cesarean section as independent risk factors for PAS [2, 17, 18, 27, 31]. In the present study, 93.97% of women with PAS and 63.59% of women with no PAS had placenta previa or low-lying placenta. Placental attachment to the lower anterior uterine wall increases the severity of PAS, especially in patients with a history of cesarean section.

The presence of placental lacunae, which appear as irregular ellipsoid shapes on ultrasound, is considered a sensitive and highly predictive indicator of PAS [32]. PAS-related placental lacunae should be differentiated from placental venous lakes, maternal blood sinuses, or liquefaction associated with placental infarction. Placental lacunae are fed by vessels that extend from the placenta across the myometrium and contain high velocity blood flow that causes turbulence on entry. Placental lacunae and their feeder vessels may be seen on color-doppler ultrasound [10, 32], which is less effective for determining blood flow in other placental spaces. In the present study, placental lacunae predicted PAS with high specificity (97.8%). Just one typical placental lacuna was found in a woman with placenta previa at 28 weeks of gestation and a woman with a left lateral wall placenta at 36 weeks of gestation. Each placental lacuna had internal blood flow signals and very thin feeding vessels extending to the uterine wall. After delivery, both cases were confirmed as placenta increta. Placental lacunae may be graded according to Finberg’s criteria [33]. Higher lacunar grade has been associated with a higher frequency and severity of PAS and is an important predictor of peripartum complications in PAS [34].

Loss of the retroplacental space and myometrial thinning as predictors of PAS have high sensitivity, low specificity and a high false-positive rate [11, 35, 36]. In the present study, loss of the retroplacental space and thickness of the retroplacental myometrium had a sensitivity and specificity of 67.5% and 78.3%, and 64.1% and 85.9%, respectively, as indicators of PAS. Importantly, subjectivity of the observer and factors such as ultrasonic beam angle, abdominal fat thickness and fullness of the bladder may affect findings related to these features on ultrasound. In a previous report, loss of the retroplacental space and myometrial thinning had excellent interobserver agreement for ultrasound imaging in the second and third trimesters [36].

Evidence suggests that subplacental hypervascularity has a low sensitivity (59%) and high specificity (95%) for PAS [37]. In contrast, in our study, subplacental hypervascularity had a sensitivity of 75.8% and specificity of 68.5% for PAS. These disparate findings may be related to differences in ultrasound instruments and observers. Previously, we have shown that subplacental blood flow velocity in the lower segment of the anterior uterine wall is higher in women with PAS compared to no PAS, with 41 cm/s as the threshold for diagnosis of PAS (sensitivity 87%, specificity 78%) [38]. Velocity is a more objective measure than color-doppler blood flow; however, this feature is only applicable when determining whether the lower anterior wall of the uterus has placental invasion.

The placenta thickens in women with PAS. A previous report showed that lower uterine segment placental thickness was significantly higher in women with an abnormally invasive placenta (AIP) and was an independent predictor of AIP [39]. In the present study, placental thickness had some significance in the diagnosis of placenta accreta, but its accuracy was low compared to other indicators. ROC curves showed little difference in placental thickness in women with no PAS, placenta accreta and placenta increta, while placental thickness changed greatly in placenta percreta.

In our study, 7 women underwent bladder repair and 3 women underwent hysterectomy. The hysterectomy rate was significantly lower than previously reported [11, 40], likely due to targeted preoperative interventional therapy. Interventions for PAS have been associated with complications [41]; however, they can reduce hemorrhage and decrease hysterectomy rates in some cases [42].

This study was associated with several limitations. First, we did not use 3D imaging. We aimed to develop a practical approach to prenatal diagnosis of PAS, and the use of 2D vs. 3D imaging may facilitate clinical uptake of our ultrasound scoring system by reducing constraints imposed by technology and cost. Notably, 3D imaging is not necessarily more accurate than 2D imaging [11, 19, 40]. Second, the false-negative rate in this study was higher than expected (30.7%), likely due to our broad inclusion criteria and the participation of multiple sonographers of different seniority. Third, gestational age as ≥ 28 weeks was selected as an inclusion criterion for this study because the ultrasound features of PAS during late pregnancy are complex but distinct. For example, placental lacunae appear as single or multiple irregularly-shaped intraplacental anechoic areas in the first or second trimester, but are larger and continuous in the third trimester. Selecting cases late in pregnancy was also conducive to accurate follow-up information. Future research will include longitudinal studies to observe changes in the characteristics of each ultrasound feature of PAS at various weeks’ gestational age, and predict the risk of PAS during the second trimester and even first trimester.

## Conclusions

In summary, this study identified seven indicators of PAS and included them in an ultrasound scoring system for PAS that has good diagnostic efficacy and clinical utility.

## Data Availability

The datasets used and/or analysed during the current study are available from the corresponding author on reasonable request.

## List of abbreviations

PAS: placenta accreta spectrum
ORs: odds ratios
95% CIs: confidence intervals
ROC: Receiver operating characteristic
AIP: Abnormally invasive placenta
